# Supplementary material for: DMRforPairs: identifying Differentially Methylated Regions between unique samples using array based methylation profiles
Source: BMC Bioinformatics. 2014 May 15;15:141. doi: 10.1186/1471-2105-15-141 (PMC4046028; doi:10.1186/1471-2105-15-141)
Supplement: Additional file 2 — DMRforPairs output for the comparison of A431-MCF7 and NA17018-NA17105. Please start from the HTML files in each folder. Available via the BMC Bioinformatics website. [file 1471-2105-15-141-S2.zip › 1394847754114233_MOESM2_ESM/A431_MCF7/figures/28305.pdf]

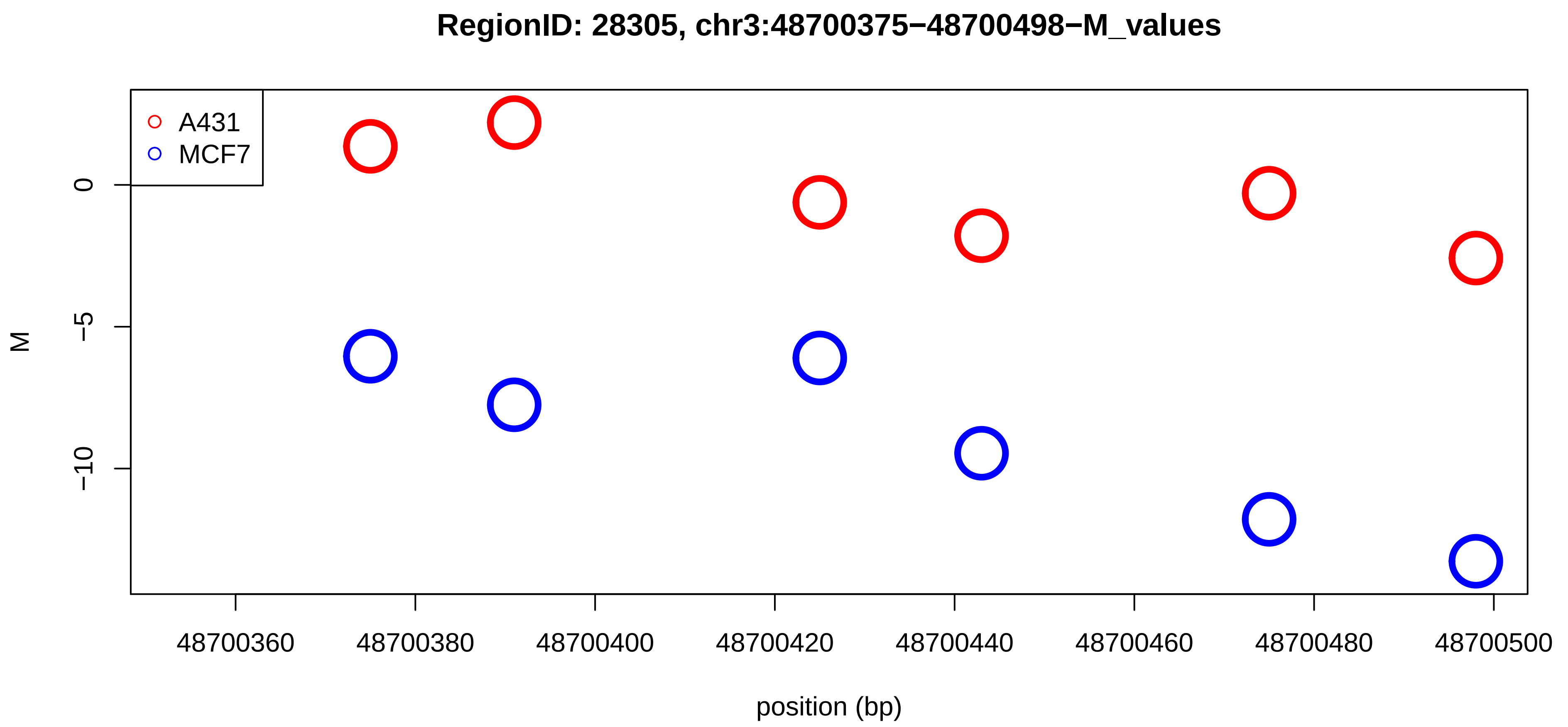
RegionID: 28305, chr3:48700375−48700498−M_values
A431
MCF7
M
0
−5
−10
48700360
48700380
48700400
48700420
48700440
48700460
48700480
48700500
position (bp)

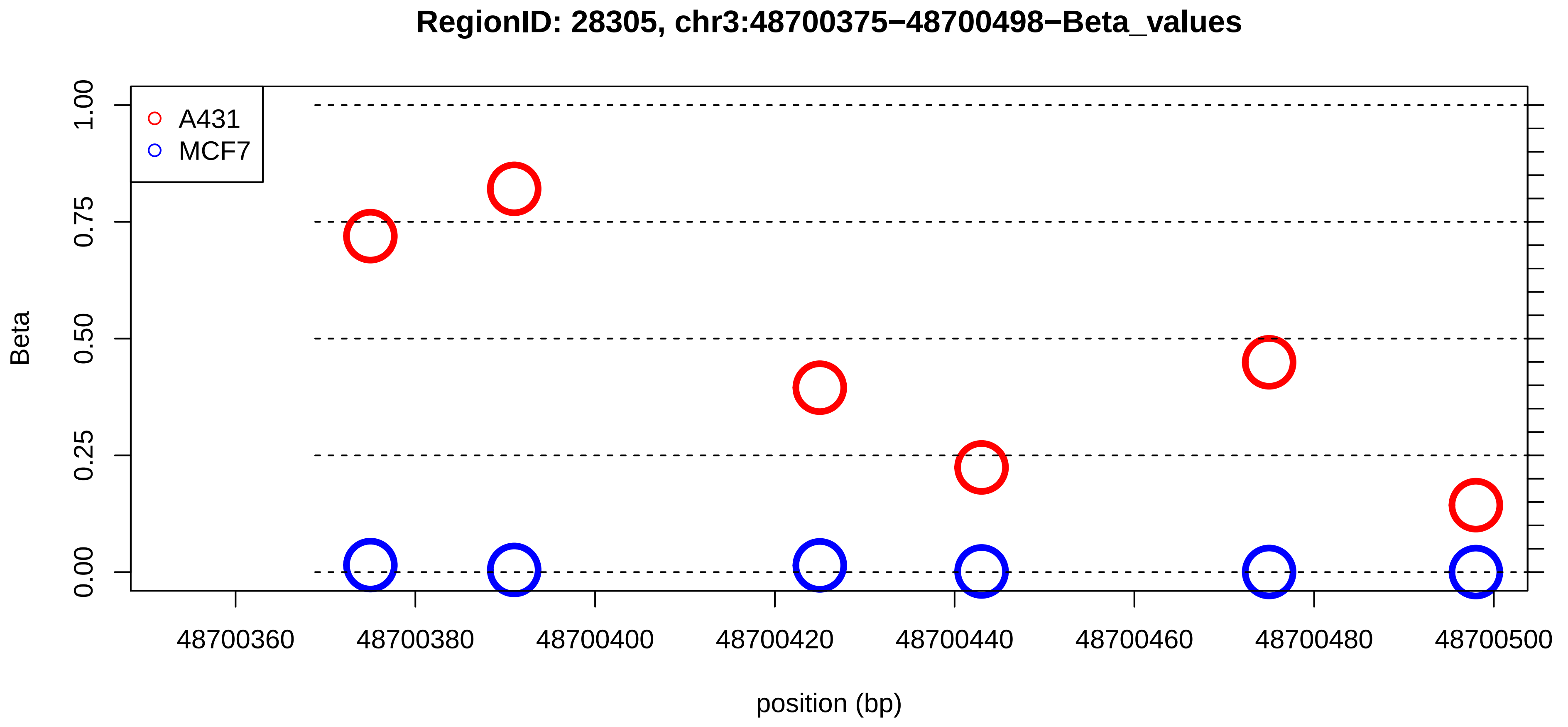
RegionID: 28305, chr3:48700375−48700498−Beta_values
A431
MCF7
Beta
0.00
0.25
0.50
0.75
1.00
48700360
48700380
48700400
48700420
48700440
48700460
48700480
48700500
position (bp)

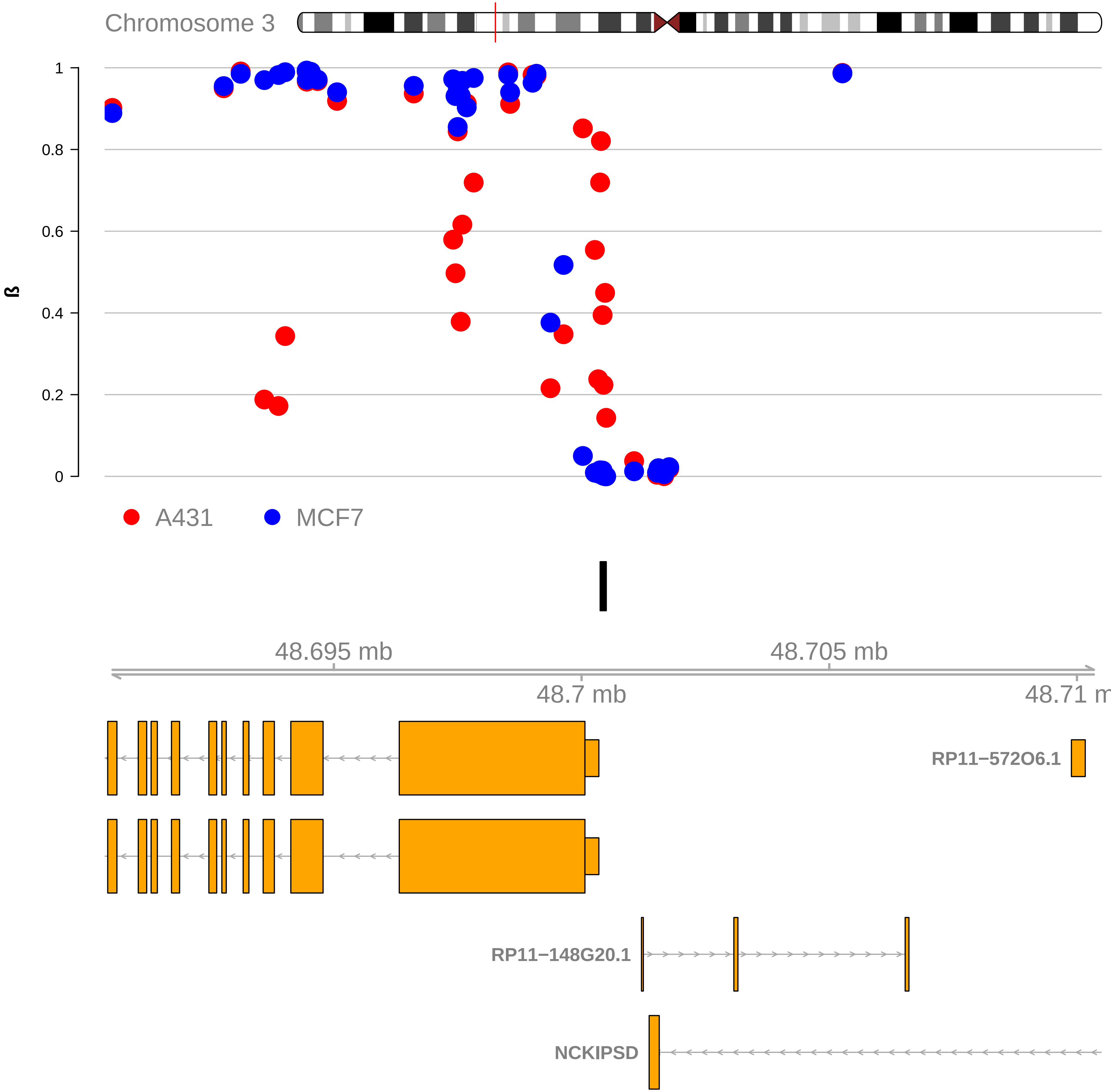
Chromosome 3
ß
1
0.8
0.6
0.4
0.2
0
A431
MCF7
48.695 mb
48.705 mb
48.7 mb
48.71 m
RP11−572O6.1
RP11−148G20.1
NCKIPSD
